# Co‐purification of nitrate reductase 1 with components of the cytochrome *bcc‐aa_3_* oxidase supercomplex from spores of *Streptomyces coelicolor* A3(2)

**DOI:** 10.1002/2211-5463.13086

**Published:** 2021-02-14

**Authors:** Dörte Falke, Marco Fischer, Christian Ihling, Claudia Hammerschmidt, Andrea Sinz, Gary Sawers

**Affiliations:** ^1^ Institute of Microbiology Martin‐Luther University Halle‐Wittenberg Halle (Saale) Germany; ^2^ Institute of Pharmacy Charles Tanford Protein Center Martin‐Luther University Halle‐Wittenberg Halle (Saale) Germany

**Keywords:** actinobacteria, nitrate respiration, O_2_ respiration, protein–protein interaction, respiratory supercomplex, spores

## Abstract

In order to reduce nitrate *in vivo*, the spore‐specific respiratory nitrate reductase, Nar1, of *Streptomyces coelicolor* relies on an active cytochrome *bcc‐aa_3_* oxidase supercomplex (*bcc‐aa_3_* supercomplex). This suggests that membrane‐associated Nar1, comprising NarG1, NarH1, and NarI1 subunits, might not act as a classical menaquinol oxidase but could either receive electrons from the *bcc‐aa_3_* supercomplex, or require the supercomplex to stabilize the reductase in the membrane to allow it to function. To address the biochemical basis for this dependence on the *bcc‐aa_3_* supercomplex, we purified two different Strep‐tagged variants of Nar1 and enriched the native enzyme complex from spore extracts using different chromatographic and electrophoretic procedures. Polypeptides associated with the isolated Nar1 complexes were identified using mass spectrometry and included components of the *bcc‐aa_3_* supercomplex, along with an alternative, spore‐specific cytochrome *b* component, QcrB3. Surprisingly, we also co‐enriched the Nar3 enzyme with Nar1 from the wild‐type strain of *S. coelicolor*. Two differentially migrating active Nar1 complexes could be identified after clear native polyacrylamide gel electrophoresis; these had masses of approximately 450 and 250 kDa. The distribution of active Nar1 in these complexes was influenced by the presence of cytochrome *bd* oxidase and by QcrB3; the presence of the latter shifted Nar1 into the larger complex. Together, these data suggest that several respiratory complexes can associate in the spore membrane, including Nar1, Nar3, and the *bcc‐aa_3_* supercomplex. Moreover, these findings provide initial support for the hypothesis that Nar1 and the *bcc‐aa_3_* supercomplex physically associate.

## Graphical abstract

Spore‐specific respiratory nitrate reductase (Nar1) uses nitrate as an electron acceptor when oxygen is limiting. Nar1 activity is totally reliant on the cytochrome *bcc‐aa_3_* oxidase supercomplex. One possibility is that the supercomplex redirects electrons to Nar1 from menaquinol (MK), as indicated by the red dotted arrow. This study reveals Nar1 copurifies with subunits of the *bcc‐aa_3_* supercomplex, supporting this hypothesis.

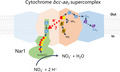

**Abbreviations**
*bcc‐aa_3_* supercomplex: menaquinol:cytochrome *c* oxidoreductase*–*cytochrome *c* oxidase supercomplex
BN‐PAGE: blue native polyacrylamide gel electrophoresis
CN/PAGE: clear native polyacrylamide gel electrophoresis
DDM: *n*‐dodecyl β‐D‐maltoside
DNB: diluted nutrient broth
MOPS: 3‐(N‐morpholino) propanesulfonic acid
MS: mass spectrometry
Nar: nitrate reductase
Q‐cycle: quinol cycle
SFM: soy flour mannitol
TSB: tryptic soy broth

## Introduction

Spores of the genus *Streptomyces* aid dispersion of the bacterium to new environments [1]; however, spores are also capable of surviving for considerable periods while remaining metabolically active [2]. Under suitable environmental conditions, spores germinate and develop into mycelium. Both spore germination and further growth as mycelium depend on respiration with molecular oxygen (O_2_) [2]. Spores are able to respire slowly with O_2_, whereby to do so they rely mainly on a proton‐translocating cytochrome *bcc‐aa_3_* oxidase supercomplex (*bcc‐aa_3_* supercomplex), comprising a diheme, quinol cytochrome *c* reductase (Qcr) complex, which is similar to mitochondrial complex III in function, and a cytochrome *c* oxidase (complex IV) of the copper *aa*_3_ type [3]. Mycelium also uses the *bcc‐aa_3_* supercomplex to facilitate energy conservation. However, when O_2_ becomes limiting, mycelium can also switch to using a high‐affinity, non‐proton‐translocating cytochrome *bd* oxidase to oxidize menaquinol directly. Spores appear to synthesize only low levels of the quinol oxidase but activate its synthesis in the complete absence of the *bcc‐aa_3_* supercomplex [4]. Endogenously produced NADH, pyruvate, and succinate, resulting from the slow degradation of the spore‐storage compound trehalose [3, 5], are assumed to function as electron donors to drive respiration.

Supercomplex formation in the respiratory chain of the high‐GC content actinobacteria is necessary because, unlike mitochondria and many oxidase‐positive bacteria that use a soluble cytochrome *c* to shuttle electrons between complexes III and IV [6], actinobacteria have a membrane‐anchored diheme cytochrome *c*, QcrC, which necessitates that complexes III and IV are in direct contact with each other for electron transfer to occur [7]. Recent studies have resolved the structure of the *bcc‐aa_3_* supercomplex from *Mycobacterium smegmatis* [8, 9], revealing it to have a linear supramolecular organization comprising a complex III (*bcc* complex) dimer flanked at each end by an *aa_3_* oxidase (complex IV). A similar structure is also proposed for the *Corynebacterium glutamicum* supercomplex [10], and our own studies have revealed that a *bcc‐aa_3_* supercomplex also exists in *S. coelicolor* [11]. However, the *bcc‐aa_3_* in *S. coelicolor* appears to have a different composition in spores compared with mycelium [11]. It is currently unclear whether this compositional difference in *S. coelicolor* is perhaps due to altered physical stability, that is, disassembly, of the streptomycete supercomplex. Notably, the cytochrome *b* (QcrB) component of the supercomplex is significantly less abundant in spores than in mycelium. It has been suggested that the expression of the gene encoding one of two other QcrB paralogs encoded in the genome of *S. coelicolor*, *qcrB3* (SCO7236), might be induced in spores and its gene product QcrB3 might be capable of functionally substituting for QrcB [11]. What advantage this would bring for spores is, however, currently unclear [3].

As well as being able to use O_2_ as an electron acceptor, *S. coelicolor* is also able to respire with nitrate [reviewed in 3]. Although respiratory nitrate reduction is incapable of supporting anaerobic growth of *S. coelicolor,* it nevertheless contributes to the maintenance of a proton motive force (*pmf*) when O_2_ becomes limiting [12]. The bacterium synthesizes three nonredundant nitrate reductases (Nar) of the membrane‐anchored NarGHI class, which reduce nitrate on the cytoplasmic side of the membrane [13]. The Nar1 enzyme is synthesized exclusively in spores and is always present and latently active, whereby activity is reversibly inhibited by O_2_ [14]. The Nar3 enzyme is mainly active in stationary phase mycelium [15], but also exhibits some weak activity in spores [16].

In a recent study, we made the unexpected discovery that manifestation of Nar1 enzyme activity in spores is completely dependent on the synthesis of an active *bcc‐aa_3_* supercomplex [17]. This unexpected and unprecedented finding is difficult to rationalize, all the more so because both transcription of the *narG1* gene and synthesis of the catalytic subunit NarG1 still occur in the mutant. Moreover, because Nar enzymes typically receive electrons directly from reduced quinone species [13], this finding suggested that Nar1 might require the *bcc‐aa_3_* supercomplex to mediate electron transfer from menaquinol to nitrate. Such a suggestion is not unprecedented, as recent studies have implicated a similar involvement of a *bcc* complex in periplasmic reduction in nitrate and nitrous oxide in the anaerobic respiration carried out by the epsilonproteobacteria *Campylobacter jejuni* and *Wollinella succinogenes* [18, 19]. These observations also pertain to a similar, earlier finding that complex III (cytochrome *bc_1_* complex) mediates electron transport to periplasmic reductases in the alphaproteobacterium *Paracoccus denitrificans* [20]. Coupling electron transfer from quinol through the electron‐bifurcating Q‐cycle of complex III [21, 22] with reduction of the nitrogen oxyanion in the periplasm would allow these bacteria to conserve energy *via* this mechanism. In the case of Nar1 in streptomycete spores, coupling a Q‐cycle mechanism with nitrate reduction in the cytoplasm would theoretically allow 4 H^+^ ions to be translocated instead of only 2 if menaquinol were to be oxidized directly by Nar1 [12]. For such a mechanism to function, however, this would require that Nar1 interacts with the *bcc‐aa_3_* supercomplex, which has never been observed. In this study, we present initial evidence through co‐purification studies, which supports the hypothesis that Nar1 indeed interacts with the *bcc‐aa_3_* supercomplex in spores of *S. coelicolor*. Moreover, we also provide evidence that Nar3 copurifies with Nar1, suggesting that respiratory enzymes might associate into large supermolecular complexes in the membrane of spores.

## Materials and methods

### Bacterial strains and culture conditions

Media and culture conditions for *S. coelicolor* and *E. coli* were the same as described [23, 24]. *S*. *coelicolor* A3(2) wild‐type strain M145, and mutant derivatives thereof, were grown on SFM (soy flour mannitol) or on DNB (diluted nutrient broth) agar [23]. Spores were prepared as described [17]. Spores were suspended either in H_2_O for use in nitrate reduction assays or in 100 mm potassium phosphate buffer pH 7.2, for disruption using a beadbeater (see below). Subsequently, spore debris and glass beads were separated from the crude extract by two centrifugation steps (4 °C, 45 000 ***g***, 5 min), as described [14].

For cultivation in liquid medium, *S*. *coelicolor* strains were grown in tryptic soy broth (TSB; Oxoid) or DNB broth supplemented as described previously [4]. Growth of *S. coelicolor* A3(2) strains as highly disperse liquid cultures in Duran‐F tubes with MOPS‐buffered TSB was performed as described [25].

*Escherichia coli* DH5α (StrataGene) was used as a host for cosmids and HST08 Stellar™ competent cells (Takara Bio) for plasmid constructions. The nonmethylating plasmid donor *E. coli* strain ET12567/pUZ8002 [26] was used for intergeneric conjugation with *S. coelicolor* [21]. Kanamycin (Kan, 25 μg ml^‐1^), chloramphenicol (Cam, 15 μg ml^‐1^), or hygromycin (Hyg, 25 μg ml^‐1^), all from Sigma, was added to growth media as required.

### Construction of strains and plasmids

The strains used in this study are listed in Table 1. Strains COE460 (Δ*qcrB3*) and COE468 (Δ*qcrB3* Δ*cydAB*) were constructed by introducing cosmid 1CO7.2.hop.EZR1.seq [27] carrying a Tn*5062* insertion in SCO7236 (*qcrB3*), into the wild‐type strain M145 and into COE190 (Δ*cydAB*) [4], respectively, and selecting for apramycin resistance and kanamycin sensitivity using already published procedures [23]. Strain COE737 was constructed by introducing the conjugative plasmid pNGnar1 [17] into M145. The genotype of the resulting mutants was confirmed by PCR.

**Table 1:** Strains and vectors used in this study

Strains	Genotype and characteristics	Reference or source
*Streptomyces coelicolor* A3(2)		
M145 (wild‐type)	SCP1^−^ SCP2^−^	[23]
NM24	M145 Δ*nar1*: ΔSCO6535–6532::*aadA* (deletion of 6209 bp removing *narG1H1J1I1*)	[14]
NM92	M145 carrying complete deletions of the *nar1, nar2,* and *nar3* operons	[16]
COE190	M145 carrying a complete deletion of the *cydAB* genes	[4]
COE192	M145 carrying a complete deletion of the *qcrCAB‐ctaE‐*SCO2152‐SCO2153‐*ctaCDF* gene locus	[4]
COE441	COE192 carrying pMS2152	[11]
COE450	COE192 carrying pMS2148‐2150	[11]
COE452	COE192 carrying pMS2148‐2151	[11]
COE454	COE192 carrying pMS2153‐2156	[11]
COE468	COE190 carrying a mutation in SCO7236::Tn*5062*	This study
COE634	COE192 carrying pMS2148‐2156	[11]
COE718	COE468 carrying pMS3945‐46	This study
COE806	NM24 carrying pMSnar1‐NarH^Strep^	This study
COE816	NM24 carrying pMSnar1‐NarI^Strep^	This study
COE818	NM92 carrying pMSnar1‐NarI^Strep^	This study
COE820	NM92 carrying pMSnar1‐NarH^Strep^	This study
COE884	COE468 carrying pNG7236^StrepC^	This study
COE886	COE468 carrying pNG7123^StrepC^	This study
*Escherichia coli*		
DH5α	F^‐^φ80*lacZ* M15 *endA recA hsdR*(r^‐^_k_m^‐^_k_) *supE thi gyrA relA*Δ(*lacZYA*‐*argF*)U169	Laboratory stock
ET12567(pUZ8002)	*dam^‐^ dcm*; with *trans‐*mobilizing plasmid pUZ8002	[26]
HST08	F^‐^, *endA1, supE44, thi‐1, recA1, relA1*, *gyrA96*, *phoA*, Φ*80d lacZΔ* M15, Δ(*lacZYA‐argF*) *U169*, Δ(*mrr‐hsdRMS‐mcrBC*), Δ*mcrA*, λ‐	Takara Bio
Plasmids and cosmid		
pMS82	ΦBT1 *attP‐int* derived integration vector for the conjugal transfer of DNA from *E. coli* to *Streptomyces* (Hyg^R^)	[29]
pMSnar1	pMS82 SCO6535–6532 (*narG1H1J1I1*)	[17]
pMSnar1‐NarH^Strep^	pMS82 SCO6535–6532 (*narH* carries codons encoding C‐terminal Strep‐tag II)	This study
pMSnar1‐NarH^Strep^	pMS82 SCO6535–6532 (*narI* carries codons encoding C‐terminal Strep‐tag II)	This study
pMS3945‐46	pMS82 SCO3945–SCO3946	[4]
pNG2	P_ermE_*, RBS, MCS, and fd‐ter cloned into pNG1’/EcoRV/SpeI Hyg^r^	[28]
pNGnar1	pNG2 carrying SCO6532–SCO6535 (*narG1H1J1I1*)	[17]
pNG7120^StrepC^	pNG2 carrying SC7120^StrepC^ including 200 bp of upstream region	This study
pNG7236^StrepC^	pNG2 carrying SC7236^StrepC^ including 74 bp of upstream region	This study
1C07.2.h09.EZR1.seq	SCO7236::Tn*5062*	[27]
Sequence of oligonucleotides used	5’ → 3’	
narI1Mut‐StrepC‐fw	GGAGCCATCCGCAGTTTGAAAAATAGCTCCCTGTTCGACTGTCC	
narI1Mut‐StrepC‐rv	ACTGCGGATGGCTCCATGCGCTGGAGCCCGCGCGCTCCCAGC	
narH1Muta‐StrepC‐fw	GGAGCCATCCGCAGTTTGAAAAATGACCCGATGCCCGGCTTCG	
narH1Muta‐StrepC‐rv	ACTGCGGATGGCTCCATGCGCTGACCTCCCTCTCGTCGTCCG	
pNG‐7120StrepC_fw	GGTGGTCATATGTCGGACGAGTCGGATTCGTCG	
pNG‐7120StrepC_rv	GGTGGTACTAGTTCATTTTTCAAACTGCGGATGGCTCCATGCGCTGGACCCGTGCCGACCGGTG	
pNG‐7236Strep_fw	GGTGGTCATATGTCCGGCAAGCCGAAGGGTGACG	
pNG‐7236Strep_rv	GGTGGTACTAGTTCATTTTTCAAACTGCGGATGGCTCCATGCGCTGCGCTC‐CGGCTCGCCCGAGTAGGC	

Plasmids pMSnar1‐NarH^Strep^ and pMSnar1‐NarI^Strep^ included the complete *narG1H1J1I1* operon, with its native promoter sequence [17], but differed in that pMSnar1‐NarH^Strep^ had additional codons on *narH* that introduced a Strep‐tag II at the C terminus of NarH and pMSnar1‐NarI^Strep^ had the same additional codons on *narI*. To construct these plasmids, pMSnar1 [17] was mutagenized by inverse PCR using the oligonucleotides (see Table 1) narI1Mut‐StrepC‐fw and narI1Mut‐StrepC‐rv to generate pMSnar1‐NarI^Strep^ and narH1Muta‐StrepC‐fw and narH1Muta‐StrepC‐rv to generate pMSnar1‐NarH^Strep^. Plasmid pNGnar1 included the complete *nar1* operon under the control of the constitutively expressed plasmid promoter present in the conjugative vector pNG2 [28], and its construction was reported previously [17]. To construct plasmids pNG7120^Strep^ and pNG7236^Strep^, the complete SCO7120 and SCO7236 genes were amplified using the oligonucleotide combinations pNG‐7120StrepC_fw/ pNG‐7120StrepC_rv and pNG7236StrepC_fw/pNG7236StrepC_rv (Table 1), respectively, the latter of which in each case included codons that introduced a Strep‐tag II at the C terminus of both gene products. Both DNA fragments were cloned in vector pNG2 using the restriction sites NdeI and SpeI. Inverse PCR was performed using PrimeSTAR GXL Polymerase (Takara Bio) according to the manufacturer’s specifications. The authenticity of the mutagenized plasmids and the insertion of the codons encoding the Strep‐tag II sequence were verified by DNA sequencing.

### Complementation of deletion mutants

Complementation studies with plasmids carrying different portions of the *qcrB‐cta* gene locus cloned in vector pMS82 [29] and involving the large SCO2148‐SCO2156 gene disruption in strain COE192 were performed as previously described [11]. All plasmids carrying genes encoding strep‐tagged Nar1, QcrB2 (SCO7120), or QcrB3 (SCO7236) were introduced into the indicated strains via conjugation using the plasmid‐containing *E. coli* strain ET12567 (pUZ8002) (Table 1), using standard procedures exactly as described previously [17].

### Protein purification

All protein purification steps were carried out aerobically and at 4 °C. Spores (between 3.5g and 10g wet weight) were suspended in 3 mL g^‐1^ spore material of buffer A (100 mm Tris/HCl, pH 8.0, containing 150 mm NaCl) for purification of Strep‐tagged proteins or of buffer B (50 mm Tris/HCl, pH 7.5) for standard purification procedures. Prior to spore disruption, 10 μg mL^‐1^ of DNase I and 0.4 mm PMSF were added to the suspension. Spores were disrupted using a beadbeater (Retsch, Hahn, Germany) by vigourous shaking for 30 min at 4 °C. A mixture of glass beads (0.4 g each of glass beads with diameters 4 mm, 1.25 mm, and 0.1 mm) was used. Subsequently, spore debris and glass beads were separated from the spore crude extract by two centrifugation steps (4°C, 45 000 ***g***, 5 min) as described [17]. Purification of Strep‐tagged Nar1 complexes was performed essentially as described in Ref. [30]. Spore crude extract was treated for 30 min at 4 °C with 15 mm *n*‐dodecyl β‐d‐maltoside (DDM) to release Nar1 from the cytoplasmic membrane. Subsequently, the detergent‐treated extract was centrifuged for 5 min at 21 500 ***g*** to remove insoluble material. To reduce binding of other biotin‐containing proteins in spores to the Strep‐Tactin^®^/XT matrix, the DDM‐solubilized crude extract was mixed with avidin (2 mm final concentration) prior to being applied to a 1 mL Strep‐Tactin^®^/XT Column (IBA Lifesciences, Goettingen, Germany), which had been previously equilibrated in buffer A containing 2 mm DDM. Crude extract was applied by gravity flow and allowed to interact with the matrix for 15 min before nonspecifically bound proteins were eluted from the column by washing with 5 column volumes of buffer A containing 2 mm DDM. Specifically bound, Strep‐tagged Nar1 complexes were eluted from the matrix by applying 3 column volumes of buffer A containing 2 mm DDM and 50 mm biotin. Fractions of 0.5 mL were collected and analyzed for Nar enzyme activity. As a negative control, a crude extract of spores derived from strain NM92 (Δ*nar1,* Δ*nar2,* Δ*nar3*) lacking a Strep‐tagged gene product was treated in exactly the same way and the eluted material was used for analysis by mass spectrometry. Polypeptides that bound nonspecifically to the affinity chromatography matrix were subtracted from those identified when the Strep‐tagged Nar1 enzyme complexes were analyzed.

Enrichment of untagged Nar1 enzyme complexes was achieved in a two‐step process. In this case, the membrane fraction was separated from spore crude extracts (38 mL) by ultracentrifugation for 1 h at 150 000 ***g*** in a Beckman Coulter Optima XPN‐80 Ultracentrifuge. The membrane vesicles were suspended in 12 mL of buffer B, to which was added 0.1 % (w/v) Triton X‐100. The mixture was incubated on a rotating wheel overnight at 4 °C. The completely solubilized membrane fraction was applied at a flow rate of 1 mL min^−1^ to a 5 mL Hi‐Trap DEAE Sepharose FF Column (Cytiva, Freiburg, Germany) pre‐equilibrated with buffer B containing 0.1% (w/v) Triton X‐100 and which was attached to an ÄKTA Protein Purifier (GE Healthcare). After collection of the flow through, nonspecifically bound proteins were washed from the column by applying 10 column volumes of buffer B containing 0.1% (w/v) Triton X‐100. Specifically bound protein complexes were subsequently eluted by applying a NaCl gradient (0–500 mm) in 50 mL of buffer B containing 0.1% (w/v) Triton X‐100. Fractions of 1 mL were collected and analyzed using the rapid, qualitative discontinuous nitrate reductase assay (see below). Fractions with Nar activity were pooled and concentrated using Vivaspin^®^ centrifugal concentrators (Sartorius, Goettingen, Germany), which had a molecular mass cutoff of 30 kDa. Subsequently, the pooled, concentrated sample (0.5 mL) was applied to a Superdex‐200‐10/300 (24 mL bed volume) column (Cytiva, Freiburg, Germany) equilibrated in buffer C (50 mm Tris/HCl, pH 7.5, 150 mm NaCl) containing 0.1% (w/v) Triton X‐100. The column was run at a flow‐rate of 0.3 mL·min^−1^, and 1 mL fractions were collected. Fractions were analyzed for Nar enzyme activity, initially qualitatively and subsequently quantitatively using the continuous BV‐based nitrate reductase assay (see below). This Nar1 enrichment procedure was performed two times, each time with an independently prepared spore suspension. Protein concentration was determined as described [31], and purified, or enriched, proteins were stored at −20 °C.

### Determination of nitrate reduction activity

Nitrate reductase enzyme activity was determined using either a discontinuous or a continuous assay. The discontinuous assay [14, 17] was used to determine nitrate reductase activity in resting spore suspensions or in column fractions. When resting spore suspensions were used, the assay was performed quantitatively and activity was determined with respect to the absorbance at 420 nm of the spore suspension, exactly as described [14, 17]. For rapid, qualitative determination of Nar activity in elution fractions after chromatographic separation of extracts, 10 μL aliquots of the column fractions were placed in 96‐well microtiter plates and 90 μL of assay buffer including 5 mm sodium nitrate was added and the samples were incubated a RT for 60 min after which time nitrite was determined by adding sulfanilamide and α‐naphthol after vigorous mixing. The absorbance change was determined colorimetrically as previously described [14, 17]. The measurement of Nar enzyme activity was also determined using the continuous assay, with reduced benzyl viologen (BV) as electron donor as described previously [14, 17]. All quantitative enzyme assays reported in this study were performed using minimally three biological and three technical replicates, unless otherwise stated.

### Polyacrylamide gel electrophoresis (PAGE) and western blotting

Gel electrophoretic analysis of native Nar enzyme complexes was done using either blue native (BN)/PAGE [11] or clear native (CN)/PAGE exactly as described in earlier studies [16, 17]. BN/PAGE was performed using gradient gels (4–16% w/v polyacrylamide) (SERVA Electrophoresis, Heidelberg, Germany) and each gel was run with molecular mass markers (SERVA Native Marker Mix), while for CN/PAGE 10% w/v polyacrylamide gels were used. Denaturing SDS/PAGE [32] used 7.5% or 12.5% w/v polyacrylamide gels, and subsequent to electrophoresis, separated polypeptides were visualized by staining either with Coomassie Brilliant Blue R or with colloidal Coomassie Blue. Alternatively, polypeptides were transferred to nitrocellulose membranes for western blot analysis, as described [32, 33]. Aliquots (typically 45–100 μg of protein) of spore crude extracts or variable volumes of chromatographically separated elution fractions were analyzed. For western blots, affinity‐purified peptide antibodies raised against NarG1 [17] were used at a dilution of 1 : 75. Secondary antibody conjugated to horseradish peroxidase was obtained from Bio‐Rad Laboratories, Feldkirchen, Germany. Visualization was done by the enhanced chemiluminescent reaction (Agilent Technologies, Waldbronn, Germany).

### In‐gel staining to detect Nar1 enzyme activity

Qualitative staining for Nar1 enzyme activity after either BN/PAGE or CN/PAGE was performed using dithionite‐reduced methyl viologen (MV) as electron donor, as described [16, 17]. Nar3 enzyme activity cannot be visualized after native PAGE [15].

### Mass spectrometry

Digestion of aliquots of the elution fractions was done according to an existing protocol [34]. Liquid chromatography/mass spectrometry (LC/MS) analysis was performed on an Ultimate 3000 RSLCnano System coupled to an Orbitrap Q‐Exactive Plus Mass Spectrometer (Thermo Fisher Scientific, Erlangen, Germany) equipped with a nano‐electrospray ionization source. The samples were loaded onto a trapping column (Acclaim PepMap C18, 300 µm × 5 mm, 5 µm, 100Å; Thermo Fisher Scientific) and washed for 15 min with 0.1 % (w/v) TFA at a flow rate of 30 µL·min^−1^. Trapped peptides were eluted using a separation column (200 cm µPAC™ C18; Pharmafluidics, Gent, Belgium) that had been equilibrated with 3 % B (A: 0.1 % (v/v) formic acid in water; B: 0.08 % (v/v) formic acid in acetonitrile). Peptides were separated with linear gradients from 3 to 8 % B (over 10 min followed by 8% B to 33% B in 80 min. The column was kept at 30°C, and the flow rate was set to 300 nL·min^−1^. MS data were collected in data‐dependent MS/MS mode during the complete gradient elution: Each high‐resolution full scan (*m/z* 375–1799, *R* = 140 000) was followed by product ion scans (HCD, higher‐energy collision‐induced dissociation, 30 % normalized collision energy) of the ten most intense signals of the full‐scan mass spectrum (isolation window 2 Th). Data analysis was performed using the Proteome Discoverer 2.3 (Thermo Fisher Scientific). MS/MS data of precursor ions (*m/z* 500–5000) were searched against the UniProt *Streptomyces coelicolor* database (version 11/2019, 8038 entries) using Sequest. Mass accuracy was set to 5 ppm and 0.02 Da for precursor and fragment ions, respectively. Carbamidomethylation of cysteine and oxidation of methionine were set as static and potential modifications, respectively, and up to two missed cleavages of trypsin were allowed. The results were filtered for proteins identified with at least two peptides with an FDR < 0.05. When protein complexes isolated by strep‐tag affinity chromatography were analyzed, identified proteins were filtered against a set of proteins identified in a spore extract fraction lacking a strep‐tagged protein, but which had otherwise been treated under identical conditions (see section 2.3 above).

## Results

### *Dependence on a functionally active* bcc‐aa_3_ *supercomplex for Nar1 enzyme activity*

**Fig. 1:**
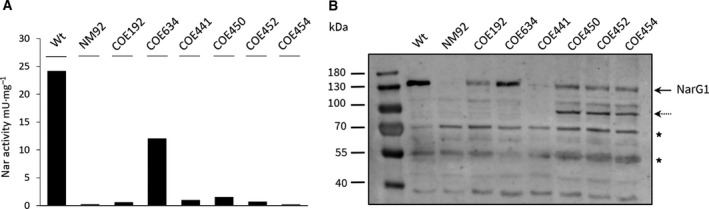
Only complementation with a conjugative plasmid carrying all genes encoding the complete *bcc‐aa_3_* supercomplex restores Nar1 activity to mutant COE192 (Δ*qcr‐cta*). (A) Specific nitrate reductase activity in crude extracts of spores after introducing plasmids carrying genes encoding different components of the supercomplex. Note that activity was measured from two independent spore preparations in each case; hence, standard deviations are not included. B. Western blot analysis of spore extracts of COE192 (Δ*qcr‐cta*) carrying partial complementation constructs of *bcc‐aa*_3_ supercomplex components. Aliquots of crude extracts of different spore extracts (65 µg) were analyzed using anti‐NarG1‐specific peptide antibodies. A thick arrow marks the signal, which indicates the migration position of NarG1; the thin arrow indicates a possible degradation product of NarG1. The asterisks mark unspecific cross‐reacting peptides that acted as protein‐loading controls. Strains from which the spores were isolated: Wt, M145; NM92 (Δ*nar1,* Δ*nar2,* Δ*nar3*); COE192 (Δ*qcr‐cta*); COE634, COE192 + pMS2148‐2156 (see Table 1 and Fig. S1); COE441, COE192 + pMS2152; COE450, COE192 + pMS2148‐2150; COE452, COE192 + pMS2148‐2151; COE454, COE192 + pMS2153‐2156 [11].

We showed recently that Nar1 enzyme activity could not be detected in resting spores of mutant COE192, which lacks the genes encoding a functional *bcc‐aa_3_* supercomplex [17]. NarG1, the catalytic subunit of Nar1, was, however, detectable in spore extracts of COE192, ruling out transcriptional control of *narG1H1J1I1* operon expression being the reason for the lack of Nar1 activity in the mutant. To investigate the dependence of Nar1 activity on the *bcc‐aa_3_* supercomplex in more detail, we first attempted to recover Nar1 activity in spores of COE192, which had been transformed with different combinations of genes from the *qcrBAC‐ctaEFDC* locus (see Fig. S1 for gene constructs used). Expression of neither *qcrBAC, qcrBACctaE,* nor *ctaEFDC* was capable of restoring Nar1 activity to spores of COE192 (Fig. 1A). Only introduction of a conjugative plasmid including the complete *qcr‐cta* gene locus was able to restore approximately 50% of the wild‐type levels of Nar1 enzyme activity to strain COE192, which also concomitantly restored cytochrome *c* oxidase activity, as reported previously [11]. Western blot analysis of the same spore extracts with α‐NarG1 antibodies revealed that NarG1 was synthesized, even in the mutant strain expressing only parts of the *qcr‐cta* gene locus, but which resulted in no recovery of Nar1 activity (Fig. 1B). However, the levels of the NarG1 polypeptides exhibited increased abundance of a presumptive degradation product (~ 50 kDa). A similar phenotype was also observed previously in spore extracts of molybdenum cofactor‐deficient mutants that also lacked Nar1 enzyme activity [16]. Together, these results indicate a strong correlation between the activity of Nar1 and the activity of the *bcc‐aa_3_* supercomplex.

### Nar1 modified by the addition of a Strep‐tag retains activity

**Fig. 2:**
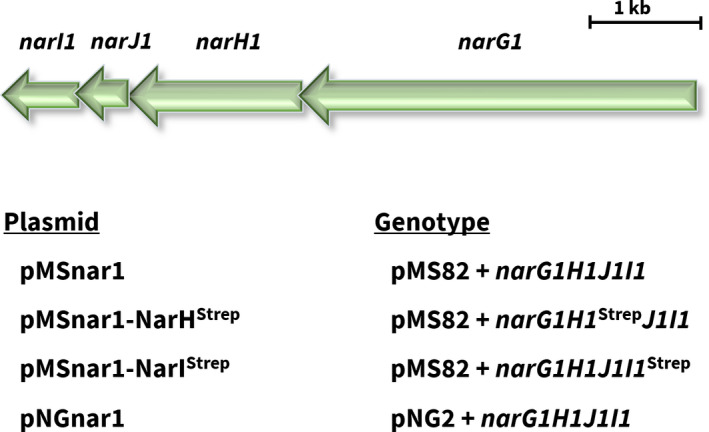
A schematic representation of the genetic locus of the operon encoding Nar1 is shown at the top of the panel. The integrative plasmids constructed and used in this study are presented below the operon.

**Fig. 3:**
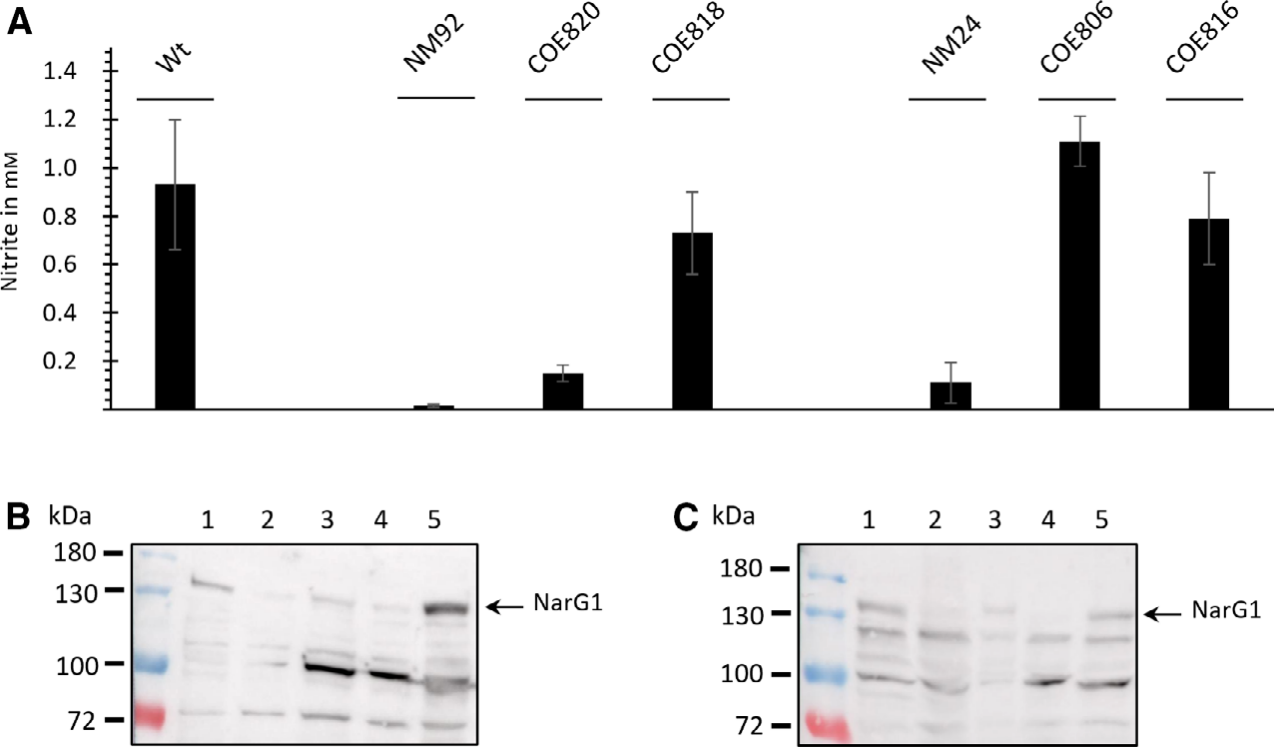
Quantitative nitrite production in resting spore suspensions by Strep‐tagged Nar1 enzyme variants. (A) Freshly prepared spore suspensions with an OD_450_ of 5 (equivalent to 1.75 × 10^9^ spores mL^−1^) were incubated in MOPS‐NaOH, pH 7, with 5 mm nitrate for 5 h under a nitrogen atmosphere. Excreted nitrite was detected in the supernatant as described [14]. The experiment shows data with standard deviation obtained from three biological replicates each performed in triplicate. Western blot analysis of mutants carrying pMSnar1‐NarH^Strep^ (B) or pMSnar1‐NarI^Strep^ (C). Aliquots (45 µg) of crude extract from the indicated strains were separated by 7.5% SDS/PAGE and transferred to nitrocellulose. Affinity‐purified anti‐NarG1 peptide antibodies [14] were used (dilution 1 : 75) to detect NarG1. An arrow marks the specific signal of NarG1. The strains included in B: lane 1, M145 (wild‐type); lane 2, NM92 (Δ*nar1,* Δ*nar2,* Δ*nar3*); lane 3, COE820 (NM92 + pMSnar1‐NarH1^Strep^; lane 4, NM24 (Δ*nar1*); and lane 5, COE806 (NM24 + pMSnar1‐NarH1^Strep^). C: lane 1, M145 (wild‐type); lane 2, NM92 (Δ*nar1,* Δ*nar2,* Δ*nar3*); lane 3, COE818 (NM92 + pMSnar1‐NarI1^Strep^; lane 4, NM24 (Δ*nar1*); and lane 5, COE816 (NM24 + pMSnar1‐NarI1^Strep^). The left‐hand lane of each gel shows the migration positions of molecular mass markers.

To investigate whether the dependence of Nar1 activity on the *bcc‐aa_3_* supercomplex might be due to a physical interaction between the complexes, we decided to isolate Strep‐tagged versions of Nar1 and to analyze the isolated complexes by mass spectrometry. It was first important to show that the addition of a C‐terminal Strep‐tag did not interfere with the manifestation of Nar1 enzyme activity in spores. To demonstrate this, two Strep‐tagged derivatives of Nar1 were made and tested. The coding sequence for the 10‐amino acid‐long Strep‐tag II (including linker) was added to the immediate 3’ end of the *narH1* and the *narI1* genes, encoding, respectively, the electron‐transferring, iron–sulfur (FeS)‐containing subunit NarH1, and the predicted heme *b*‐containing, membrane anchor subunit NarI1 of the Nar1 enzyme (Fig. 2). To determine whether the addition of the Strep‐tag interfered with assembly or activity of the Nar1 enzyme, the two integrative plasmids pMSnar1‐NarH^Strep^ and pMSnar1‐NarI^Strep^ (Table 1) were introduced into the genome of strains NM24 (Δ*nar1*) and NM92 (Δ*nar1,* Δ*nar2,* Δ*nar3*), which carry single or multiple deletions in the operons encoding Nar1, Nar2, and Nar3 [14]. Spores of strains carrying integrative plasmid pMSnar1‐NarH^Strep^ or pMSnar1‐NarI^Strep^ (see Table 1) were then prepared, and their ability to reduce nitrate to nitrite was determined (Fig. 3A). Spores of NM24 (lacking spore‐specific Nar1) reduced nitrate at a level that was approximately 90% lower compared with the activity in wild‐type spores. The residual activity in spores of NM24 is mainly due to activity of Nar3 [14]. Introduction of pMSnar1‐NarH^Strep^ (strain COE806) restored nitrate‐reducing activity to levels slightly higher than those of M145, while introduction of pMSnar1‐NarI^Strep^ into NM24 (COE816) restored activity to levels slightly lower than wild‐type (Fig. 3A). These data demonstrate that the addition of the Strep‐tag to the NarH1 and NarI1 subunits in this genetic background did not affect the capacity of Nar1 to reduce nitrate *in vivo*.

Nitrate‐reducing activity of spores of NM92 (Δ*nar1,* Δ*nar2,* Δ*nar3*) without plasmid was essentially undetectable in spores of NM92 (Fig. 3A). The measurement of nitrite production after introduction of both plasmids into NM92 revealed that pMSnar1‐NarI^Strep^ (COE818) complemented the Nar1‐negative phenotype effectively, while Nar1 carrying a Strep‐tag on NarH1 (COE820) restored activity only poorly (Fig. 3A). These results suggest interdependence between the Nar enzymes and that introduction of the C‐terminal Strep‐tag on the NarH subunit either impairs electron transfer to the Nar1 enzyme or possibly interferes with correct assembly of the enzyme.

To determine whether Nar1 was stably synthesized when either NarH1 or NarI1 carried a C‐terminal Strep‐tag, the catalytic subunit NarG1 of the enzyme was visualized immunologically in extracts of spores derived from strains NM24 (Δ*nar1*) and NM92 (Δ*nar1,* Δ*nar2,* Δ*nar3*) each transformed with either pMSnar1‐NarH^Strep^ (Fig. 3B) or pMSnar1‐NarI^Strep^ (Fig. 3C). The results demonstrate that all the complemented strains synthesized an approximately 130 kDa NarG1 polypeptide, which is close to its deduced molecular mass of 135.8 kDa. Notably, the levels of the NarG1 polypeptide in the spore extracts of the respective strains correlated with the levels of nitrate reduction detectable in the strains. This suggests that NarG1 levels were lower in the NM92 background lacking the other Nar enzymes, due perhaps to an altered stability of the Nar1 complex in the membrane.

### Purification of proteins associated with NarH^Strep^ and NarI^Strep^

**Fig. 4:**
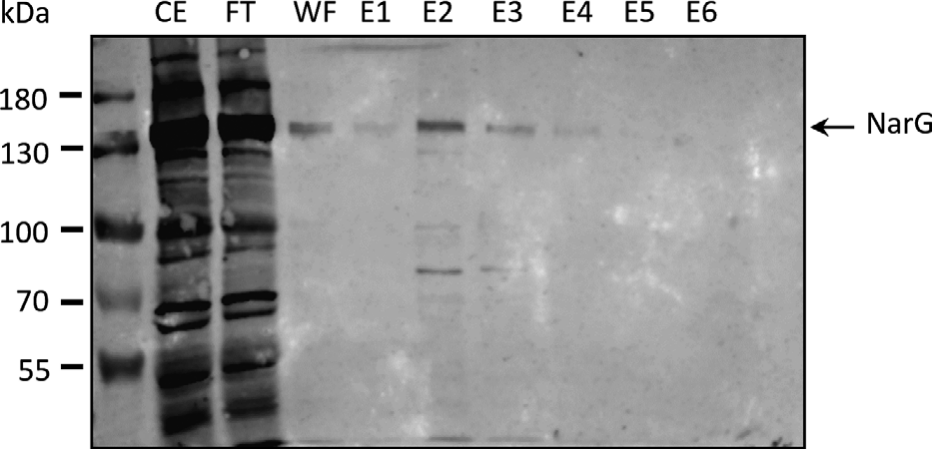
Western blot analysis of protein extracts and elution fractions after NarI^Strep^ purification. Crude extract (CE), flow through (FT), wash fraction (WF), and elution fractions (E1‐E6) of the NarI1^Strep^ purification (from spores of COE818) were separated by 7.5% SDS/PAGE and transferred to nitrocellulose. NarG1 was detected using affinity‐purified peptide antibodies anti‐NarG1 (1:75). An arrow marks the specific signal of NarG1 migrating at approximately 135 kDa.

Due to the limited amount of Nar1 enzyme present in spores, together with the apparently variable stability of the enzyme activity, we initially wished to determine whether Nar1 could be isolated as an intact and active enzyme. As a secondary challenge, and because of the complete dependence for Nar1 enzyme activity on the *bcc‐aa_3_* supercomplex, we also wished to determine whether any other proteins relevant to the supercomplex could be co‐purified with either the membrane anchor subunit, NarI1^Strep^, or with the electron‐transferring FeS‐containing NarH1^Strep^ subunit. To do this, spores from COE818 (NM92 + pMSnar1‐NarI^Strep^) and COE820 (NM92 + pMSnar1‐NarH^Strep^) were isolated, and after disruption and detergent treatment to release Nar1 from the membrane fraction [14], the Strep‐tagged polypeptides were purified by Strep‐Tactin^®^ Affinity Chromatography. Aliquots of the fractions eluted from the column after affinity purification of NarI1^Strep^ from spores of COE818 (NM92 + pMSnar1‐NarI^Strep^) were initially analyzed by western blotting using antiserum specific for NarG1 (Fig. 4). The blot revealed that the NarG1 polypeptide could be detected mainly in elution fractions E2 and E3. These data indicate that the heterotrimeric Nar1 enzyme was assembled and could be isolated from the membrane fraction using NarI1^Strep^, because NarG1 is attached via NarH to NarI [35].

Unfortunately, only low amounts of NarG1 could be detected after affinity chromatographic enrichment using both Strep‐tagged derivatives, which was likely due to the generally low abundance of total protein present in the elution fractions (e.g., E2 contained 100–150 μg total protein). Therefore, to identify Nar polypeptides and any others that co‐purified with the NarH^Strep^ and NarI^Strep^ subunits, we analyzed samples of the peak elution fraction E2 or E3 (see Fig. 4) from the samples derived from spores of COE818 (NM92 + pMSnar1‐NarI^Strep^) and COE820 (NM92 + pMSnar1‐NarH^Strep^) by mass spectrometry. As a control to rule out nonspecific binding of proteins to the affinity matrix, the affinity purification experiment was repeated under identical conditions using a spore extract derived from strain NM92 lacking a Strep‐tagged protein. The polypeptides identified in the E2 or E3 elution fraction of this purification were used to filter the list of polypeptides identified after the NarH^Strep^ and NarI^Strep^ affinity purification experiments. The complete lists of identified polypeptides that were identified to be copurified with each Strep‐tagged subunit are presented as supplementary files (Tables S1 and S2). Due to the biochemical dependence for Nar1 activity on the *bcc‐aa_3_* supercomplex, polypeptides which are either part of Nar1 or are relevant to the composition of the *bcc‐aa_3_* supercomplex, and which were identified to co‐elute, are summarized in Table 2. As anticipated, based on the findings of the Nar enzyme activity and western blot analyses (see Figs. 3 and 4), NarG1 and NarH1 were identified after both purifications. While purification of NarI^Strep^ identified both NarI1 and the private chaperone of Nar1, NarJ1, neither of these proteins was identified in the elution fraction derived from the purification of NarH^Strep^ (Table 2). The latter finding supports the observation that Nar1 shows variable stability in spore extracts. Moreover, the identification of bound NarJ1 in the NarI^Strep^ sample suggests that a population of this Nar1 enzyme was incompletely, or was in the process of being, assembled [36].

**Table 2:** Co‐eluting peptides identified by mass spectrometry to interact with NarH^Strep^ and NarI^Strep^. Only polypeptides identified to be related to the *bcc‐aa_3_* supercomplex and nitrate reductase are shown^a^

SCO number^b^	Enzyme/protein	Description	Peptides^c^	PSMs	Mass [kDa]
SCO2148	QcrB	Cytochrome *b* component	1	5	60.8
SCO2151	CtaE	Probable cytochrome c oxidase subunit III	1	3	22.7
*SCO6532	NarI1	Putative nitrate reductase gamma chain NarI	4	4	26.9
*SCO6533	NarJ1	Putative nitrate reductase chaperone NarJ	1	1	18.4
SCO6534	NarH1	Putative nitrate reductase beta‐chain NarH	6	15	59.7
SCO6535	NarG1	Putative nitrate reductase alpha‐chain NarG	21	41	135.8
SCO7236	QcrB3	Probable cytochrome *b* subunit	1	2	61.4

^a^ For a complete list of the copurifying polypeptide identified, see supplementary files Table S1 and Table S2

^b^ The gene products marked with an asterisk were only identified after purification of the NarI1^Strep^ protein; otherwise, all other polypeptides that were identified were copurified in both purifications.

^c^ Number of identified peptides and peptide‐spectrum matches (PSM).

Interestingly, the same three polypeptides co‐purified with both Strep‐tagged Nar1 subunits and included the cytochrome *b* subunit, QcrB, and the cytochrome *aa_3_* oxidase subunit III, CtaE from the cytochrome *bcc‐aa_3_* supercomplex, and QcrB3 (SCO7236), which is paralogous to QcrB and is proposed to be specifically synthesized in spores [3].

### Co‐purification of native Nar1 and Nar3 and components of the *bcc‐aa_3_* supercomplex

**Fig. 5:**
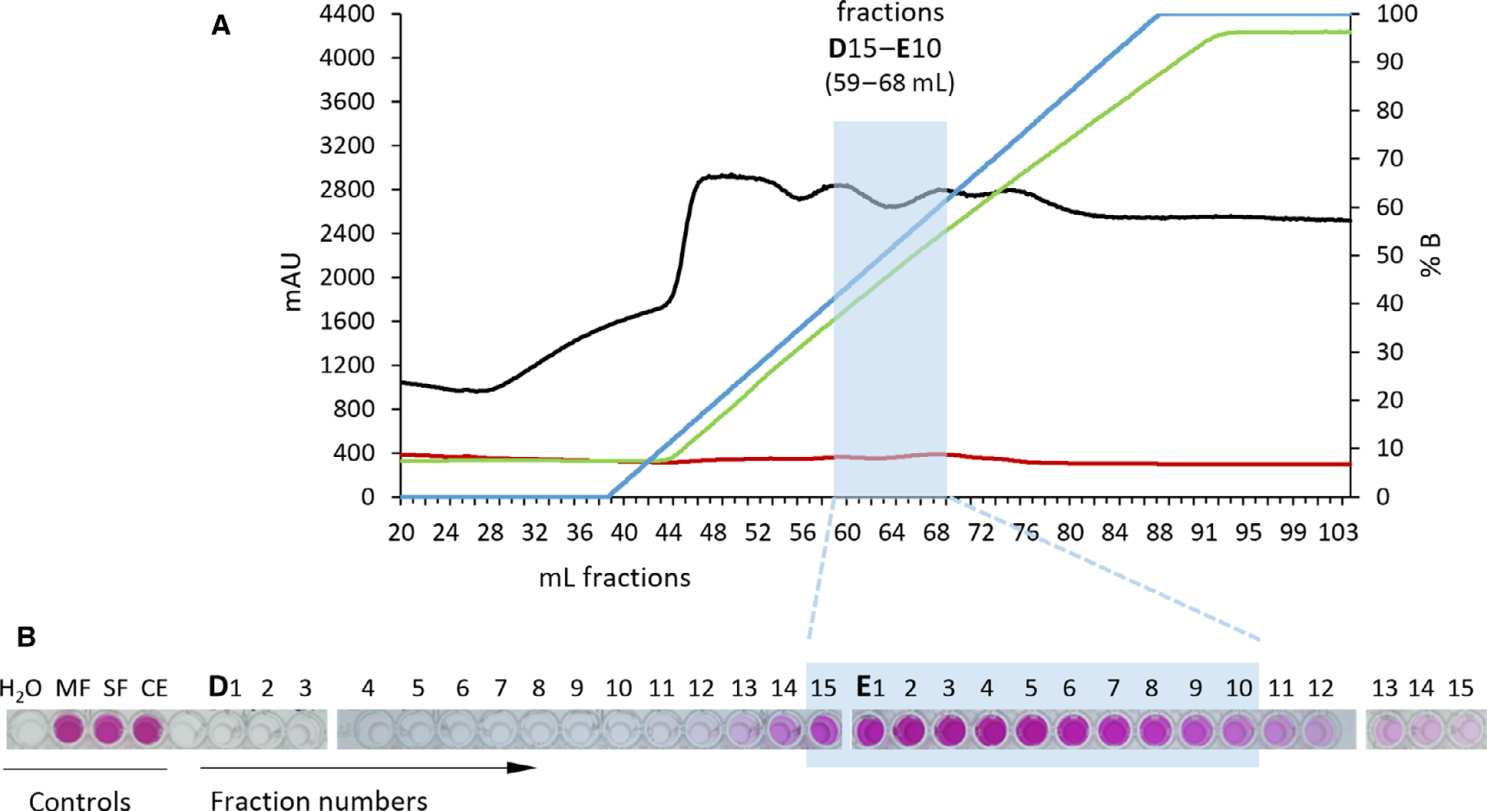
Enrichment of native Nar1 by anion‐exchange chromatography. A. The elution profile after DEAE column chromatography is shown. The light‐blue rectangle highlights the elution fractions that contained Nar enzyme activity and which were pooled and concentrated for analysis by size‐exclusion chromatography. The NaCl gradient is shown in percent with 100% being equivalent to 500 mm NaCl. Note that due to 0.1% (w/v) Triton X‐100, the buffer has an inherent background absorbance at 280 nm (black line). The ionic strength is shown as conductivity (green line) and as percentage concentration of B (blue line). The red line signifies absorbance at 420 nm. B. The results of the rapid nitrate reduction assay are shown below the elution profile. Aliquots of elution fractions D1‐D15 (equivalent to fractions 45 to 59 in part A) and E1‐E15 (equivalent to fractions 60 to 74 in part A) were analyzed in the discontinuous nitrate reductase assay. Nitrite production is visible as a purple color change. MF, spore membrane fraction; SF, spore soluble fraction; CE, spore crude extract.

**Fig. 6:**
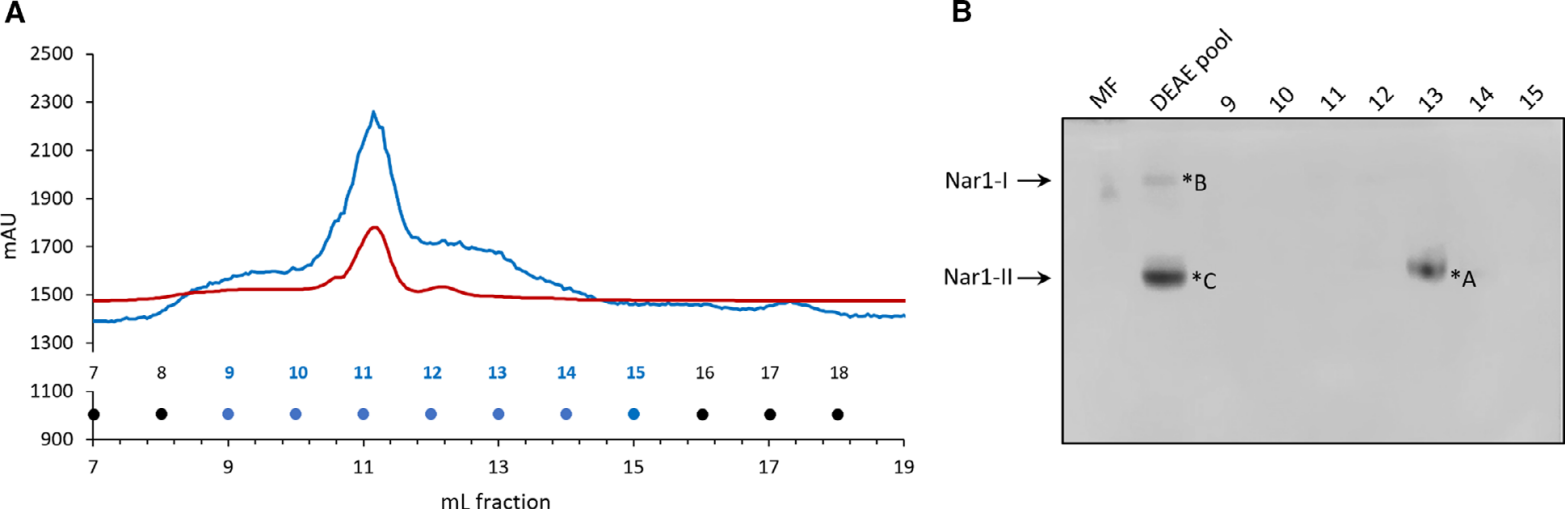
Chromatographic separation of the DEAE pool by size exclusion on a Sephadex‐200 column. (A) The elution profile after size‐exclusion chromatography is shown whereby fractions of 1 mL were collected. The absorbance changes at 280 nm (blue line) and 420 nm (red line) were monitored. The exclusion volume of the column was 8.4 mL. Nar enzyme activity was detected in fraction 13. (B) In‐gel activity staining of Nar1 complexes after CN/PAGE. Aliquots of 20 µL of membrane fraction (MF), 10 µL of the pooled concentrated DEAE fraction (DEAE pool), and 20 µL of the elution fractions after size‐exclusion chromatography (9–15) were analyzed by in‐gel nitrate reductase activity staining (see Materials and Methods). A negative of the achromatic zone in the blue‐stained gel reveals Nar1 activity in fraction 13. The arrows signify two activity bands labeled Nar1‐I and Nar1‐II. Note that Nar3 enzyme activity cannot be detected after CN/PAGE.

Having shown that Strep‐tagged Nar1 co‐purified with components of the *bcc‐aa_3_* supercomplex, we decided to determine the significance of this finding by enriching the native enzyme from the membrane fraction of spores using a different detergent and employing different chromatographic procedures. Moreover, to increase the amount of Nar1 enzyme available for analysis, we introduced into the parental strain M145 an extra copy of the *narG1H1J1I1* operon, expressed from a constitutive promoter on the integrative plasmid pNGnar1 [17]. Solubilized Nar1 was first bound to DEAE sepharose and eluted using an increasing NaCl gradient (Fig. 5A). Nar enzyme activity eluted between approximately 200 and 350 mm NaCl (Fig. 5B), and the indicated 10 fractions were pooled and concentrated to a final volume of 0.5 mL as described in the Materials and Methods. The pooled fractions containing Nar1 activity were chromatographed by size exclusion on a Sephadex‐200 Column (Fig. 6A). The absorbance traces at 280 nm and 420 nm indicated separation of polypeptides within the sample. There was both a strong 420 nm absorbance peak at approximately 300–350 kDa (fraction 11), separate from a weak absorbance peak (fraction 12) at around 200 kDa (based on migration of molecular mass marker proteins; data not shown).

**Fig. 7:**
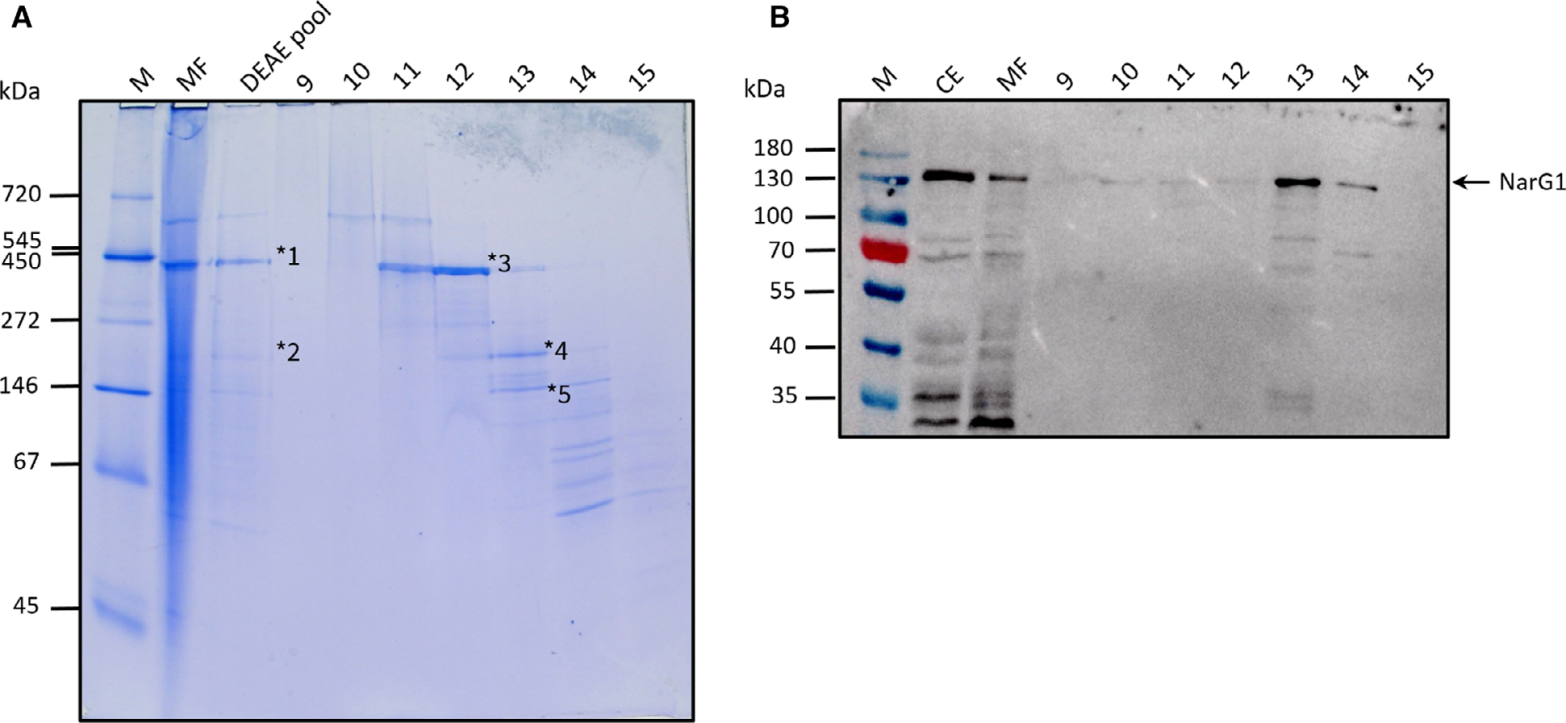
Gel electrophoretic analysis of elution fractions after Sephadex‐200 size‐exclusion chromatography. A. Aliquots of elution fractions (20 µL) were separated by BN/PAGE (4–16% polyacrylamide), and the gel was stained with Coomassie Brilliant Blue. Fractions included the membrane fraction (MF; 50 µg pf protein), the pooled DEAE fraction (DEAE pool), and elution fractions 9–15. The sizes of molecular mass markers (M) are indicated on the left in kDa. The asterisks labeled 1–5 signify protein bands that were excised and analyzed by mass spectrometry. (B) Western blot analysis of elution fractions after size‐exclusion chromatography. Aliquots (50 µg protein) of crude extract (CE), membrane fraction (MF), and 20 µL of elution fractions after size‐exclusion chromatography (9–15) were separated by denaturing SDS/PAGE (10% w/v polyacrylamide) and analyzed to detect for the presence of the NarG1 polypeptide using anti‐NarG1‐specific peptide antibodies. An arrow on the right of the panel indicates the migration position of NarG1.

Analysis of an aliquot of each elution fraction from the size‐exclusion column by CN/PAGE revealed a single active Nar1 species in fraction 13 (labeled Nar1‐II in Fig. 6B). Notably, a second, slower‐migrating and weakly active enzyme species with Nar activity (Nar1‐I in Fig. 6B) could also be discerned in the concentrated, pooled sample from the DEAE column (lane labeled DEAE pool in Fig. 6B). Unfortunately, these active enzyme species proved to be inactive after BN/PAGE (data not shown); however, staining of the gel with Coomassie Brilliant Blue after BN/PAGE revealed that fraction 13 had two weakly staining protein complexes, one of which (band 4) migrated with an approximate molecular mass of 250 kDa and the other (band 5) with a mass of 150 kDa (Fig. 7A). Aliquots of the elution fractions were also separated by SDS/PAGE and western blotting with anti‐NarG1 antibodies confirmed the presence of Nar1 in fraction 13 (Fig. 7B). Traces of NarG1 were also detected in fractions 10 through 12.

The three activity bands labeled A, B, and C in Fig. 6B were isolated from the gel and analyzed by mass spectrometry, and the complete list of polypeptides identified is included in Table S3. Those polypeptides relevant to nitrate and oxygen respiratory complexes are listed in Table 3. All three bands included the NarG1 and NarH1 polypeptides of the Nar1 enzyme. The NarG1 polypeptide was particularly abundant in the band isolated from fraction 13 (band A). Surprisingly, all the isolated bands also included NarG3 and NarH3 polypeptides of the Nar3 enzyme; however, these were present in lower abundance compared with NarG1 and NarH1 (Table 3). Migrating at the same positions as Nar1 and Nar3 were five polypeptide components of the *bcc‐aa_3_* supercomplex, including QcrB (SCO2148), QcrA (SCO2149), CtaD (SCO2155), CtaC (SCO2156), and CtaE (SCO2150). The slower‐migrating band B also included the alternative cytochrome *b*, QcrB3 (SCO7236), and the catalytic subunit of the cytochrome *bd* oxidase, CydA.

**Table 3:** Peptides and PSMs identified by mass spectrometry to co‐purify with native Nar1 chromatography after in‐gel activity staining. Only polypeptides identified to be related to the *bcc‐aa_3_* supercomplex and nitrate reductase are shown^a^

Sample	SCO number	Enzyme/protein	Description	Peptides	PSMs	Mass [kDa]
A	SCO6534	NarH	Putative nitrate reductase beta‐chain NarH	17	55	59.7
	SCO6535	NarG	Putative nitrate reductase alpha‐chain NarG	52	178	135.8
	SCO4947	NarG3	Nitrate reductase alpha‐chain NarG3	13	24	135
	SCO4948	NarH3	Nitrate reductase beta‐chain NarH3	6	12	59.8
	SCO2156	CtaC	Probable cytochrome c oxidase subunit 2	2	3	35.4
	SCO2149	QcrA	Cytochrome bc1 complex Rieske iron–sulfur subunit	3	6	38.6
	SCO2150	QcrC	Cytochrome bc1 complex cytochrome c subunit	1	1	27.5
B	SCO6534	NarH	Putative nitrate reductase beta‐chain NarH	6	18	59.7
	SCO6535	NarG	Putative nitrate reductase alpha‐chain NarG	30	49	135.8
	SCO4947	NarG3	Nitrate reductase alpha‐chain NarG3	18	38	135
	SCO4948	NarH3	Nitrate reductase beta‐chain NarH3	2	4	59.8
	SCO2156	CtaC	Probable cytochrome c oxidase subunit 2	11	56	35.4
	SCO2155	CtaD	Probable cytochrome c oxidase subunit 1‐alpha	9	38	64.1
	SCO2149	QcrA	Cytochrome bc1 complex Rieske iron–sulfur subunit	6	30	38.6
	SCO2148	QcrB	Cytochrome bc1 complex cytochrome b subunit	7	21	60.8
	SCO2150	QcrC	Cytochrome bc1 complex cytochrome c subunit	3	6	27.5
	SCO7236	QcrB3	Ubiquinol‐cytochrome C reductase cytochrome subunit B	5	11	61.4
	SCO3945	CydA	Putative cytochrome oxidase subunit I	3	5	55.9
C	SCO6534	NarH	Putative nitrate reductase beta‐chain NarH	21	69	59.7
	SCO6535	NarG	Putative nitrate reductase alpha‐chain NarG	52	163	135.8
	SCO4947	NarG3	Nitrate reductase alpha‐chain NarG3	14	33	135
	SCO4948	NarH3	Nitrate reductase beta‐chain NarH3	6	12	59.8
	SCO2156	CtaC	Probable cytochrome c oxidase subunit 2	2	5	35.4
	SCO2149	QcrA	Cytochrome bc1 complex Rieske iron–sulfur subunit	5	11	38.6
	SCO2150	QcrC	Cytochrome bc1 complex cytochrome c subunit	1	3	27.5

^a^ For a complete list of the copurifying polypeptide identified, see supplementary file Table S3.

The two protein complexes identified in fraction 13 by Coomassie staining after BN/PAGE of the fractions from the size‐exclusion column (Fig. 6A), along with two species (bands 1 and 2) from the pooled DEAE fraction (Fig. 7A), and the major protein species that migrated with a mass of 400 kDa in fraction 12 (band 3) (Fig. 7A) were carefully excised from a gel run in parallel but stained with colloidal Coomassie Blue. These isolated, stained bands were analyzed by mass spectrometry. The complete list of identified polypeptides for each band is included in supplementary Table S3, and polypeptides relevant to nitrate and oxygen respiration are listed in Table 4. A similar pattern of polypeptides from Nar1 and Nar3 and from the *bcc‐aa_3_* supercomplex was identified (Table 4). In contrast to the activity‐stained CN/PAGE, QcrB3 was identified in the faster‐migrating band of ~ 250 kDa in the pooled DEAE fraction, and was also a component of the major protein complex in fraction 12 migrating at ~ 450 kDa, which also included significant amounts of the *bcc‐aa_3_* supercomplex subunits. These results confirmed, using a different purification protocol and without use of a Strep‐tag, that components of the Nar1 and the *bcc‐aa_3_* supercomplex co‐purified. The NarG3 and NarH3 polypeptides of the Nar3 enzyme were also confirmed by this experiment to co‐purify with the same complexes. Finally, examination of the complete list of identified proteins (Table S3) also identified 13 different polypeptides associated with complex I (Nuo—NADH dehydrogenase) and seven different polypeptides of the ATP synthase. Together, these data suggest that different respiratory complexes might associate with each other in the spore membrane.

**Table 4:** Peptides and PSMs identified by mass spectrometry to co‐purify with native Nar1 chromatography after BN/PAGE. Only polypeptides identified to be related to the *bcc‐aa_3_* supercomplex and nitrate reductase are shown^a^

Sample	SCO number	Enzyme/protein	Description	Peptides	PSMs	Mass [kDa]
1	SCO6534	NarH	Putative nitrate reductase beta‐chain NarH	3	3	59.7
	SCO6535	NarG	Putative nitrate reductase alpha‐chain NarG	13	24	135.8
	SCO4947	NarG3	Nitrate reductase alpha‐chain NarG3	5	7	135
	SCO4948	NarH3	Nitrate reductase beta‐chain NarH3	2	2	59.8
	SCO2156	CtaC	Probable cytochrome c oxidase subunit 2	3	4	35.4
	SCO2155	CtaD	Probable cytochrome c oxidase subunit 1‐alpha	3	6	64.1
	SCO2149	QcrA	Cytochrome bc1 complex Rieske iron–sulfur subunit	4	12	38.6
	SCO2148	QcrB	Cytochrome bc1 complex cytochrome b subunit	4	9	60.8
2	SCO6534	NarH	Putative nitrate reductase beta‐chain NarH	5	7	59.7
	SCO6535	NarG	Putative nitrate reductase alpha‐chain NarG	22	35	135.8
	SCO4947	NarG3	Nitrate reductase alpha‐chain NarG3	5	9	135
	SCO4948	NarH3	Nitrate reductase beta‐chain NarH3	2	2	59.8
	SCO2156	CtaC	Probable cytochrome c oxidase subunit 2	20	7	35.4
	SCO2155	CtaD	Probable cytochrome c oxidase subunit 1‐alpha	6	11	64.1
	SCO2149	QcrA	Cytochrome bc1 complex Rieske iron–sulfur subunit	3	8	38.6
	SCO2148	QcrB	Cytochrome bc1 complex cytochrome b subunit	3	7	60.8
	SCO2150	QcrC	Cytochrome bc1 complex cytochrome c subunit	1	2	27.5
3	SCO6534	NarH	Putative nitrate reductase beta‐chain NarH	2	2	59.7
	SCO6535	NarG	Putative nitrate reductase alpha‐chain NarG	11	20	135.8
	SCO4947	NarG3	Nitrate reductase alpha‐chain NarG3	1	2	135
	SCO4948	NarH3	Nitrate reductase beta‐chain NarH3	1	2	59.8
	SCO2156	CtaC	Probable cytochrome c oxidase subunit 2	2	3	35.4
	SCO2155	CtaD	Probable cytochrome c oxidase subunit 1‐alpha	1	2	64.1
	SCO2149	QcrA	Cytochrome bc1 complex Rieske iron–sulfur subunit	4	8	38.6
	SCO2148	QcrB	Cytochrome bc1 complex cytochrome b subunit	3	7	60.8
4	SCO6534	NarH	Putative nitrate reductase beta‐chain NarH	12	24	59.7
	SCO6535	NarG	Putative nitrate reductase alpha‐chain NarG	41	97	135.8
	SCO4947	NarG3	Nitrate reductase alpha‐chain NarG3	13	26	135
	SCO4948	NarH3	Nitrate reductase beta‐chain NarH3	6	9	59.8
	SCO2156	CtaC	Probable cytochrome c oxidase subunit 2	4	6	35.4
	SCO2149	QcrA	Cytochrome bc1 complex Rieske iron–sulfur subunit	4	9	38.6
	SCO2148	QcrB	Cytochrome bc1 complex cytochrome b subunit	2	5	60.8
5	SCO6534	NarH	Putative nitrate reductase beta‐chain NarH	5	7	59.7
	SCO6535	NarG	Putative nitrate reductase alpha‐chain NarG	6	8	135.8
	SCO4947	NarG3	Nitrate reductase alpha‐chain NarG3	1	2	135
	SCO4948	NarH3	Nitrate reductase beta‐chain NarH3	3	7	59.8
	SCO2156	CtaC	Probable cytochrome c oxidase subunit 2	2	3	35.4
	SCO2149	QcrA	Cytochrome bc1 complex Rieske iron–sulfur subunit	3	9	38.6
	SCO2148	QcrB	Cytochrome bc1 complex cytochrome b subunit	1	2	60.8

^a^ For a complete list of the copurifying polypeptide identified, see supplementary file Table S3.

### Cytochrome *b* paralogue QcrB3 causes Nar1 to shift into the high‐molecular mass complex during CN/PAGE

**Fig. 8:**
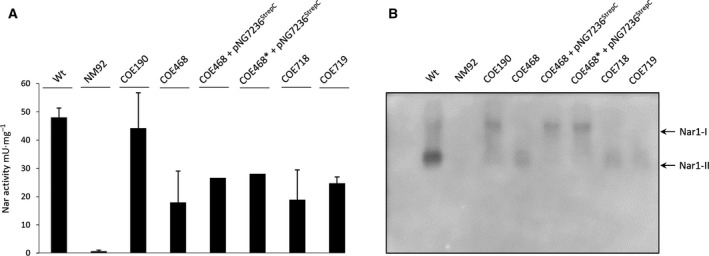
Effect of QcrB3 and cytochrome *bd* oxidase on Nar1 complex migration. (A) Specific nitrate reductase activity was determined in crude extracts prepared from spores of the indicated strains. Error bars represent standard error of the mean. (B) In‐gel activity staining of Nar1 after CN/PAGE (10% w/v polyacrylamide) is shown in negative image mode, where Nar1 enzyme activity is signified by a darkened band. Active protein complexes in spore crude extracts (45 µg protein) derived from the indicated strains were analyzed. Two different complexes (Nar1‐I and Nar1‐II) exhibiting activity are indicated on the right‐hand side of the panel.

While lack of the *bcc‐aa_3_* supercomplex abolishes Nar1 activity, deletion of the genes encoding the high‐affinity cytochrome *bd* oxidase was shown to cause a mild reduction in Nar enzyme activity in spores [17] (Fig. 8A). The copurification of the alternative cytochrome *b* component, QcrB3, with the Nar1 and Nar3 and *bcc‐aa_3_* supercomplexes, suggests that it might be able to substitute for QcrB (SCO2148) in the supercomplex in spores. Deletion of the SCO7236 gene, which encodes QcrB3, had no effect on Nar1 enzyme activity in spores of the mutant (data not shown). However, when the mutation was combined with a *cydAB* mutation (strain COE468 in Fig. 8A), Nar enzyme activity was decreased by approximately 50% in spores of the mutant when compared with the activity in spores of COE190 (Δ*cydAB*) (Fig. 8A). Re‐introduction of a gene encoding a Strep‐tagged variant of QcrB3 resulted in only partial recovery of Nar1 activity (Fig. 8A). However, when Nar1 enzyme activity was visualized after separation of spore extracts in CN/PAGE, the presence of Strep‐tagged QcrB3 in the spores caused Nar1 activity to appear mainly in the slower‐migrating complex labeled Nar1‐I (Fig. 8B). To demonstrate that this was reproducible, we tested a second, independently prepared spore extract in which the gene encoding Strep‐tagged QcrB3 was expressed in the COE190 background and the same result was observed (Fig. 8B). As a further control to demonstrate that this effect was specific, we introduced a conjugative plasmid carrying a gene encoding a Strep‐tagged variant of a second QcrB paralog, QcrB2 (SCO7021), into strain COE190 and this failed to cause a shift in the migration of Nar1 enzyme activity from the faster‐migrating Nar1‐II complex to the slower‐migrating complex, Nar1‐I (Fig. S2). This result rules out that the Strep‐tag was responsible for this effect and demonstrates that the shift in mobility observed with active Nar1 was specific. Finally, we noted that while deletion of the gene encoding cytochrome *bd* oxidase caused the slower‐migrating active Nar1‐I complex to predominate after CN/PAGE, additional deletion of *qcrB3* (strain COE468) caused Nar1 activity to be mainly present in the faster‐migrating Nar1‐II complex (Fig. 8B). Together, these findings indicate that there is a strong correlation between the presence of QcrB3 and an alteration in the mobility of Nar1 and suggest that QcrB3 affects association of Nar1 with different respiratory complexes in spores of *S. coelicolor*.

## Discussion

The findings of this study provide the first evidence that subunits of the Nar1 enzyme in spores of *S. coelicolor* can be co‐purified with components of the *bcc‐aa_3_* supercomplex, suggesting a possible interaction between these respiratory complexes. Notably, two different Strep‐tagged subunits of the Nar1 enzyme identified the same two potential interaction partners as being the alternative of the two cytochromes *b*, QcrB3, and subunit III of the cytochrome oxidase, CtaE (see also Fig. S1). This is the first demonstration that QcrB3, which is located at a different position on the genome compared with the co‐localized *qcr‐cta* genes encoding the *bcc‐aa_3_* supercomplex [11], is synthesized [3]. QcrB (SCO2148) and QcrB3 (SCO7236) share 55% amino acid sequence identity and 70% overall similarity and although it has not yet been unequivocally demonstrated that QcrB3 forms an alternative *bcc‐aa_3_* supercomplex, replacing QcrB, the fact that it was identified by mass spectrometry to be in the slow‐migrating Nar1‐I complex (see Figs. 6B and 7A) suggests that it might indeed be part of the supercomplex in spores.

As an independent demonstration that Nar1 co‐purifies with other respiratory complexes, we decided to attempt to enrich the Nar activity from the membrane fraction of wild‐type spores using conventional chromatographic separation techniques. After separation by anion‐exchange and size‐exclusion chromatographies, and adopting two different electrophoretic separation methods, we could clearly show that the catalytically active Nar1 enzyme co‐purified not only with several components of the *bcc‐aa_3_* supercomplex, but also with the catalytic and electron‐transferring subunits of the Nar3 enzyme. The results presented both in the current study and in a previous study have shown that Nar3 is weakly active in spores [16, 17]. Moreover, in the absence of Nar3, the Nar1 enzyme was less active in spore extracts, which led to the suggestion that perhaps the Nar3 enzyme somehow ‘stabilizes’ Nar1 [16]. Unfortunately, conditions have not yet been found that allow the activity of the Nar3 enzyme to be detected after gel electrophoretic separation [15]; however, the suggestion that Nar3 might help to stabilize Nar1 could explain firstly the fact that Nar1 and Nar3 copurifiy and secondly why both the activity and amount of Strep‐tagged Nar1 (NarH^Strep^) were low in spores of NM92, which lacks Nar2 and Nar3. The biochemical basis for this hypothetical stabilization of Nar1 will require further study, but it also might underlie our previous observations that Nar1 is susceptible to enzyme activity loss, independently of whether anoxic conditions are maintained or not, and this is also commensurate with a possible disassembly of the enzyme [17].

During this study, we have also shown that Nar1 migrates during native gel electrophoresis as two distinct and active species, the faster‐migrating species having a mass of around 250 kDa, while the slower‐migrating species has a mass of around 450 kDa. The trimeric Nar1 species including NarG1, NarH1, and NarI1 has a predicted mass of around 215 kDa (190 kDa without NarI1). This suggests that the 450 kDa species is possibly either a dimer of trimers or a form in which Nar1 associates with other membrane complexes, for example, with Nar3. A *bcc‐aa_3_* supercomplex exhibiting oxidase activity in spores migrates in BN/PAGE with a mass of around 270 kDa [11], which would be compatible with a total mass of around 450 kDa for a Nar1‐*bcc‐aa_3_* supercomplex. Our demonstration that by introducing strep‐tagged QcrB3 into strain COE468 (Δ*cydAB, qcrB3*) caused a shift of Nar1 activity to the 450 kDa complex also supports the hypothesis that Nar1 might interact with such a supercomplex species. Notably, our demonstration that re‐introduction of only some of the genes encoding components of the *bcc‐aa_3_* supercomplex into COE192 (Δ*qcr‐cta*) failed to restore Nar1 enzyme activity suggests that only the complete and active *bcc‐aa_3_* supercomplex allows manifestation of Nar1 activity.

If Nar1 indeed interacts with a form of the *bcc‐aa_3_* supercomplex in the membrane of spores, then this would be in accord with our previous suggestion [12] that electrons from the diheme *c*‐type cytochrome QcrC could potentially be diverted to heme *b*_D_ of NarI1. This might also be the reason for the dependence of Nar1 activity on the presence of an active *bcc‐aa_3_* supercomplex. Due to the fact that we could rule out transcriptional or translational control of *nar1* operon expression as being the reason for the reliance of Nar1 on the supercomplex [17; this study], the most straightforward interpretation of this dependence is that Nar1 does not function as a classical quinol oxidase but requires the *bcc‐aa_3_* supercomplex to mediate electron transfer from menaquinol. Such a dependence on the *bcc‐aa_3_* supercomplex could have a potentially beneficial consequence for the bioenergetics of spores if Nar1 can accept electrons from the *bcc* complex while still allowing the Q‐cycle to function, essentially replacing the *aa_3_* oxidase. The redox potentials of the redox cofactors within the *bcc‐aa_3_* supercomplex from *C. glutamicum* have been determined [10] (see Fig. S3), and the redox potentials of the redox cofactors for the Nar enzyme from *E. coli* are also known [37]. While there might well be differences in some of the potentials of the cofactors within the Nar1 enzyme of *S. coelicolor*, these potentials nevertheless suffice to serve as a model to indicate that coupling of Nar1 with the Q‐cycle is in principle feasible. Assuming involvement of the dual cytochromes *c* of QcrC, electron transfer directly to the heme *b*_D_ and heme *b*_P_ pair within NarI1 would be on a similar redox potential level as transfer to the Cu_A_ center in CtaC of the terminal oxidase. With a redox potential difference (Δ*E*) between menaquinol (*E*_o_’ = −75 mV) and the NO_3_^−^/NO_2_^−^ couple (*E*_o_’ = +430 mV) of approximately 505 mV under standard conditions, coupled with the fact that NO_3_^−^ reduction occurs in the cytoplasm, this should be sufficient to allow the Q‐cycle to move 4 H^+^ ions against a proton motive force of ~ 150 mV [38]. Nar enzymes do not pump protons, which is why 4 H^+^ ions would be translocated rather than the usual 6 H^+^ ions translocated when O_2_ acts as acceptor, that is, 4 H^+^ ions via the Q‐cycle plus a further 2 H^+^ ions via the proton‐pumping cytochrome *c* oxidase [6]. Nevertheless, translocation of 4 H^+^ ions when nitrate is used as acceptor would be 2 H^+^ ions more that would be translocated compared to when Nar acts directly as a quinol oxidase [35]. Consequently, the more efficient energy coupling of the Q‐cycle to NO_3_^−^ reduction would be highly beneficial to the persistence of spores in environments where O_2_ becomes limiting or during anaerobiosis [39].

While we cannot make any statement yet about whether Nar1 is only transiently associated with the *bcc‐aa_3_* supercomplex in spores, or what the stoichiometry of the interaction might be, based on the low abundance of Nar1 relative to the *bcc‐aa_3_* supercomplex in spores [11, 14], we believe the composition of this complex could be flexible and variable depending on electron acceptor availability, which is supported by our electrophoretic analysis of the active Nar1 complexes in the membrane of spores revealed by the present study. Moreover, it is also currently unclear why Nar1 does not appear to function as a quinol oxidase, even when it is constitutively expressed in mycelium [17]. It is also currently unclear how the Nar1 enzyme interacts with the supercomplex and what makes it different from other Nar enzymes. The NarI membrane anchor subunits of Nar enzymes are variable in amino acid sequence conservation [3, 13], making functional predictions highly speculative. Future experiments, including a detailed chemical cross‐linking analysis, will be necessary to provide an answer to these questions.

Finally, our findings of a co‐purification of Nar1 with subunits of the *bcc‐aa_3_* supercomplex support the findings of recent studies revealing that *W. succinogenes* and *C. jejuni* both show a dependence on a cytochrome *bcc* complex for periplasmic reduction in nitrous oxide or nitrate/trimethylamine‐*N*‐oxide, respectively [18, 19]. In contrast to the situation with streptomycete spores, acceptor reduction in these epsilonproteobacteria occurs in the periplasm. Coupling the energetically less‐efficient periplasmic reduction of these toxic anions to the Q‐cycle would nevertheless allow 2 H^+^ ions to be translocated per electron pair instead of none if menaquinol were to be directly oxidized at the periplasmic side of the membrane by the terminal reductase. Collectively, these findings suggest that coupling a Q‐cycle mechanism within a cytochrome *bcc* complex to a terminal reductase might be more common in anaerobic respiration than previously considered, which also has obvious relevance to persistence in certain pathogenic bacteria, such as *M. tuberculosis* [40]. The findings of our study provide a first biochemical indication that such a mechanism might indeed be feasible and furthermore are consistent with the existence in streptomycete spores of a large supramolecular respiratory network, perhaps analogous to what has been observed in *Pseudomonas aeruginosa* [41].

## Conflict of interest

The authors declare no conflict of interest.

## Author contributions

DF, MS, AH, CI, and CH carried out all the experiments. DF, MF, AS, and RGS designed the experiments and analyzed the data. RGS drafted the manuscript and conceived the study. All authors read and approved the final manuscript.

## Supporting information


**Fig. S1.** Schematic representation of the plasmid constructs used to test restoration of Nar1 activity to strain COE192 (Δ*qcr‐cta*).
**Fig. S2.** The presence of QcrB2 (SCO7120) does not influence the migrations mobility of the Nar1 complexes.
**Fig. S3.** Schematic representation of the plasmid constructs used to test restoration of Nar1 activity to strain COE192 (Δ*qcr‐cta*).

**Table S1.** Detailed list of MS data.

**Table S2.** Detailed list of MS data.

**Table S3.** Detailed list of MS data.

## Data Availability

All data presented in this paper will be made available upon request by contacting the corresponding author.
