# Supplementary material for: Co‐purification of nitrate reductase 1 with components of the cytochrome bcc‐aa3 oxidase supercomplex from spores of Streptomyces coelicolor A3(2)
Source: FEBS Open Bio. 2021 Feb 14;11(3):652–69. doi: 10.1002/2211-5463.13086 (PMC7931247; doi:10.1002/2211-5463.13086)
Supplement: Supplementary file 1 — Fig. S1. Schematic representation of the plasmid constructs used to test restoration of Nar1 activity to strain COE192 (Δqcr‐cta). Fig. S2. The presence of QcrB2 (SCO7120) does not influence the migrations mobility of the Nar1 complexes. Fig. S3. Schematic representation of the plasmid constructs used to test restoration of Nar1 activity to strain COE192 (Δqcr‐cta). [file FEB4-11-652-s001.pdf]

Co-Purification of Nitrate Reductase 1 with Components of the Cytochrome *bcc-aa$_3$* Oxidase Supercomplex from Spores of *Streptomyces coelicolor* A3(2)

Dörte Falke[1], Marco Fischer[1], Christian Ihling[2], Claudia Hammerschmidt[1], Andrea Sinz[2], and R. Gary Sawers[1*]

[1] Institute of Microbiology, Martin-Luther University Halle-Wittenberg, Kurt-Mothes-Str. 3, 06120 Halle (Saale), Germany

[2] Institute of Pharmacy, Charles Tanford Protein Center, Martin-Luther University Halle-Wittenberg, Kurt-Mothes-Str. 3a, 06120 Halle (Saale), Germany

[#] Address correspondence to: R.G. Sawers, Institute for Biology/Microbiology, Martin-Luther University Halle-Wittenberg, Kurt-Mothes-Str. 3, 06120 Halle (Saale) Germany; phone +49 345 5526350; Fax. +49 345 5527010; Email gary.sawers@mikrobiologie.uni-halle.de.

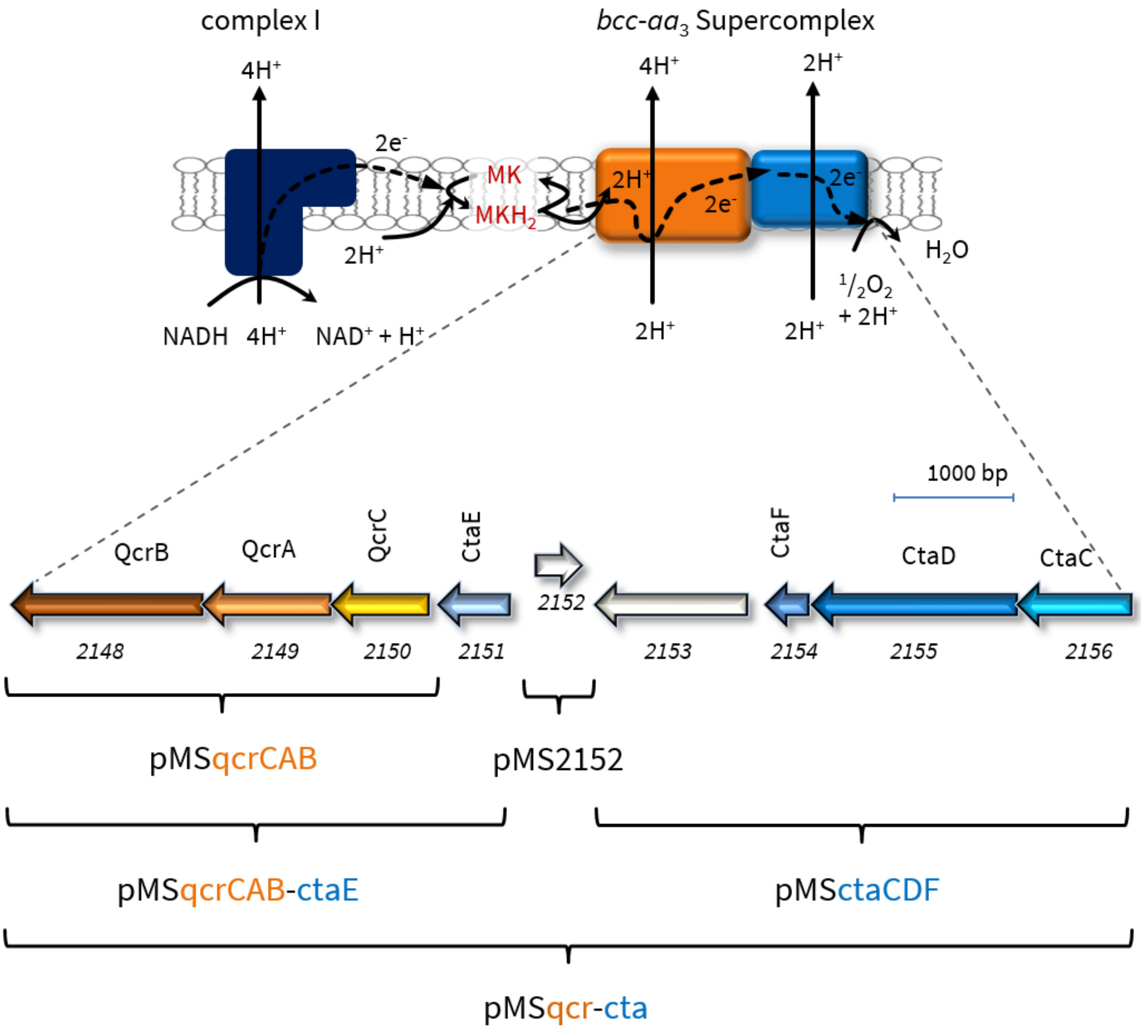

Figure S1. Schematic representation of the plasmid constructs used to test restoration of Nar1 activity to strain COE192 (Δ*qcr-cta*). The genes encoding the cytochrome *bcc* and *aa3* oxidase complexes of *S. coelicolor* A3(2) are shown with the SCO number placed under the respective gene and the product of the respective gene depicted above it. The genes encoding the cytochrome *bcc* complex and the *aa3* oxidase are colored brown and blue, respectively. The products of the genes shown in grey do not contribute of oxygen respiration or to nitrate respiration [1]. The extent of the DNA fragments cloned in the respective complementation plasmids is shown below the locus.

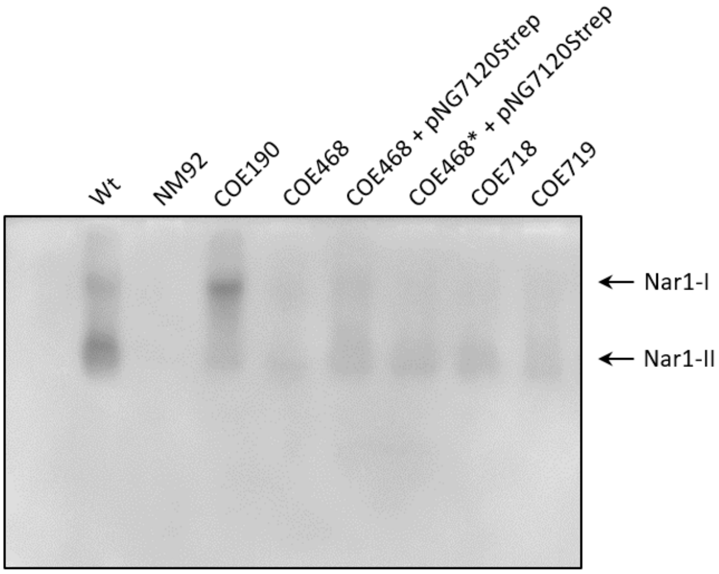

Figure S2. The presence of QcrB2 (SCO7120) does not influence the migrations mobility of the Nar1 complexes. In-gel activity staining of Nar1 after CN-PAGE (10% w/v polyacrylamide) is shown in negative image mode. Protein complexes in aliquots (45 µg protein) of spore crude extracts were analyzed. Two different complexes (Nar1-I and Nar1-II) exhibiting activity are indicated on the right hand side of the panel. Strains: Wt, M145; NM92 (Δ*nar1, 2, 3*); COE190 (Δ*cydAB*); COE468, COE190 (*qcrB2*::Tn*5062*); COE718 and COE719, two version of COE468 carrying pMS3945-3946 (*cydAB*$^{+}$).

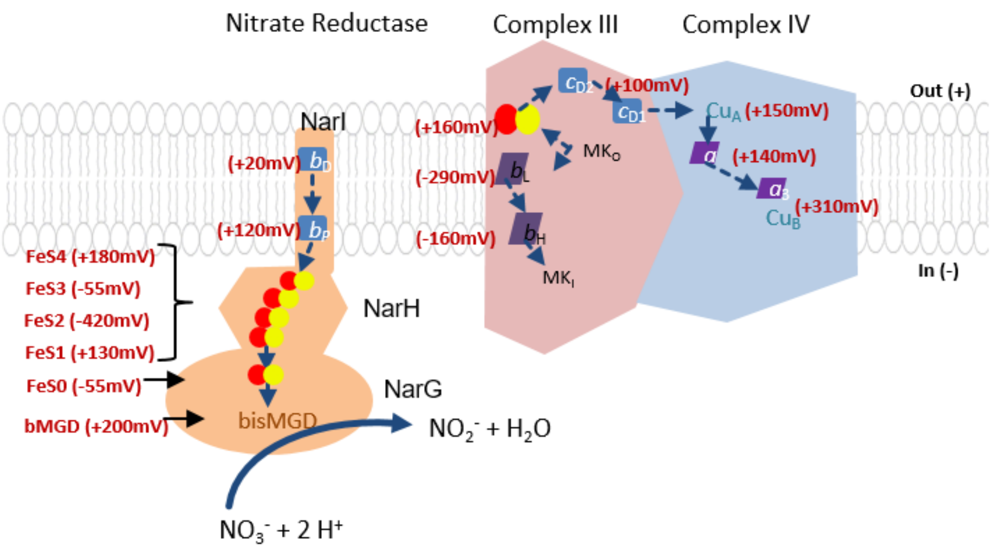

Figure S3. Schematic model of the redox cofactors in the *bcc-aa₃* supercomplex and Nar1. The redox potentials for Nar1 are predicted and are based on those determined for the *E. coli* enzyme [2] and those for the *bcc-aa₃* supercomplex are based on the complex from *C. glutamicum* [3]. Only one half of the *bcc-aa₃* supercomplex is drawn in the interest of clarity and it is predicted that NarI1 aligns at the interface of complex III (red) and IV (blue) allowing electron transfer from cytochrome c_{D1} (small blue rectangle) in QcrC to heme b_D (small brown rectangle) in NarI1 to occur. FeS clusters are represented red and yellow spheres, while hemes *a* are shown as purple rectangles.

References.

1 Falke, D, Fischer, M, Biefel, B, Ihling, C, Hammerschmidt, C, Reinefeld, D, Haase, A, Sinz, A, and Sawers, RG (2018) Cytochrome *bcc-aa₃* oxidase supercomplexes in the aerobic respiratory chain of *Streptomyces coelicolor* A3(2). *J Mol Microbiol Biotechnol* **28**: 255-268

2 Bertero, MG, Rothery, RA, Boroumand, N, Palak, M, Blasco, F, Ginet, N, Weiner, JH & Strynadka, NCJ (2005) Structural and biochemical characterization of a quinol binding site of *Escherichia coli* nitrate reductase A. *J Biol Chem* **280**: 14836–14843

3 Kao, WC, Kleinschroth, T, Nitschke, W, Baymann, F, Neehaul, Y, Hellwig, P, Richers, S, Voncke, J, Bott, M, & Hunte, C (2016) The obligate respiratory supercomplex from *Actinobacteria*. *Biochim Biophys Acta* **1857**: 1705-1714
